# Standard Precautions and Infection Control, Medical Students’ Knowledge and Behavior at a Saudi University: The Need for Change

**DOI:** 10.5539/gjhs.v5n4p114

**Published:** 2013-04-21

**Authors:** Tarek Tawfik Amin, Khalid Ibrahim Al Noaim, Mohammed Ahmed Bu Saad, Turki Ahmed Al Malhm, Abdullah Abdulaziz Al Mulhim, Marwah Abdulaziz Al Awas

**Affiliations:** 1 Public Health, Faculty of Medicine, Cairo University, Cairo, Egypt; 2 Family and Community Medicine, College of Medicine, King Faisal University, Hofuf, Saudi Arabia; 3 College of Medicine, King Faisal University, Hofuf, Saudi Arabia

**Keywords:** standard precautions, infection control, knowledge, medical students, Saudi Arabia

## Abstract

**Background:**

No previous studies have reported the knowledge of Saudi medical students about Standard Precautions (SPs) and infection control.

**Objectives:**

The objectives of this study were to assess medical students’ knowledge in clinical years at King Faisal University, Saudi Arabia about SPs’ and to explore their attitudes toward the current curricular/training in providing them with effective knowledge and necessary skills with regard to SPs.

**Subjects and Methods:**

This cross sectional study targeted students in clinical stage at College of Medicine, King Faisal University, Saudi Arabia. A pre-tested anonymous self administered data collection form was used. Inquires about students’ characteristics, general concepts of infection control/SPs, hand hygiene, personal protective equipment, sharp injuries and disposal, and care of health providers were included. The main source of information for each domain was also inquired. The second part dedicated to explore the attitudes toward the curricular and teaching relevant to SPs.

**Results:**

A total of 251 students were included. Knowledge scores in all domains were considerably low, 67 (26.7%) students scored ≥ 24 (out of 41points) which was considered as an acceptable level of knowledge, 22.2% in 4_th_ year, 20.5% in 5_th_ year and 36.8% in 6_th_ year. Sharp injuries, personal protective equipment and health care of the providers showed the least knowledge scores. The main sources of knowledge were self learning, and informal bed side practices The majority of students’ believed that the current teaching and training are insufficient in providing them with the necessary knowledge and skills regarding SPs.

**Conclusion:**

The overall knowledge scores for SPs were low especially in the domains of hand hygiene, sharp management, and personal protective equipment reflecting insufficient and ineffective instructions received by medical students through the current curriculum posing them vulnerable to health facilities related infections. Proper curricular reform and training are required to protect students and their patients.

## 1. Introduction

Standard precautions (SPs) are designed to reduce the risk of acquiring occupational infection from both known and unexpected sources in the healthcare setting (Seigel et al., 2007). Strict adherence by health staff (healthcare providers [HCPs] including students) to SPs may prevent a percentage of these risks (Philips & Ker, 2006). SPs have two objectives namely to protect HCPs from percutaneous injuries and to prevent transmission of nosocomial infection. Due to their limited experience in performing invasive procedures, medical students are at particular risk of exposure to blood-borne pathogens (Jeffe et al., 1907; Wiwanikit, 2002). Medical students should have adequate knowledge before their initial training period at hospital which is a pre-requisite for compliance. Moreover, Elliott et al. reported that specialized training must be received before a health care student undertakes any patient procedure involving sharp devices (Elliot et al., 2005). Physicians’ knowledge of SPs has been reported to be insufficient (Sax et al., 2005; Bryce et al., 2007; Easton et al., 2007). Few studies (Askarian et al., 2004; Mann & Wood 2006; Koeing & Chu, 1993; Elliot et al., 2005) have reported on medical students’ knowledge of SPs or sharp injuries and noted a lack of adequate knowledge of SPs. The observance of hygiene recommendations by students is reported as being weak: medical students rarely wash their hands after examining patients (Feather et al., 2000; Hunt et al., 2005). Poor compliance may have its roots in a failure to learn this simple, essential behavior at medical school (Feather et al., 2005). Learning practices are indispensable for improving student knowledge of nosocomial infection (Kim et al., 2001; Calabro et al., 1998) and the prevention of infection transmission (Colombo et al., 2002). In Saudi Arabia, it was reported that there was a lack of knowledge and compliance of infection control measures by health care providers in hospitals as well as at primary level of care (Maqbool, 2002). This was partially explained by the deficiency of the curricular content of medical and nursing schools in Saudi Arabia (Maqbool, 2002) as well as in many other developing countries where the role of SPs and infection control is not emphasized and SPs are often practiced incompletely, with limited understanding and thus suboptimal compliance (Maqbool, 2002; Kermode et al., 2005). Assessing medical students’ knowledge towards SPs will aid in prevention of nosocomial infections and can provide the foundations for curricular reform necessary to provide them with adequate knowledge and skills.

Studies form Saudi Arabia that assessed medical students’ knowledge towards SPs are scarce. The objectives of this study were to assess medical students’ knowledge in clinical years at King Faisal University, Saudi Arabia about SPs’ and to explore their attitudes toward the current curricular/training in providing them with effective knowledge and necessary skills with regard to SPs.

## 2. Subjects and Methods

### 2.1 Setting and Design

This was a cross-sectional study carried out at College of Medicine, King Faisal University, Saudi Arabia. The college is running two curricula concurrently, traditional and problem based learning (launched in 2012). The total number of students enrolled (both genders) were 435 in the clinical years (2_nd_ semester of the 4_th_, 5_th_ and 6_th_ years) and about 500 in the preclinical stage. The current traditional curriculum has no course (partially or in whole) that is dedicated for orientation/training of medical students in regard infection control and SPs, and relevant information are delivered through scattered lectures in the context of other courses namely microbiology and epidemiology in the preclinical stage.

### 2.2 Subjects

All medical students at the College of Medicine, King Faisal University of both genders at their clinical rotations were invited to participate. Data collection has taken place between April 1_st_ and June 20_th_, year 2012.

### 2.3 Data Collection

Data were collected through a self administered anonymous questionnaire including the following sections:

-Socio-demographics including age, year of study at the college, gender, received previous training or educational materials about infection control and SPs. The average number of patients’ the medical students were exposed to in the last 30 days, hand washing frequency following exposure to patients’.
-Knowledge about different domains of infection control and SPs: consisted of closed ended questions in multiple choice or the options of true, false and I do not know format. The used items were adopted from the available literature (Amin & Al Wehedy, 2009; Tavolacci et al., 2008); the English version of the questionnaire was used as it is the formal teaching language. The questionnaire included quires about the different domains of SPs namely the general concepts of infection control and SPs (5 questions), hand hygiene (10 questions), personal protective equipment (PPE) (9 questions), sharps disposal and injuries (8 questions) and care of health care providers to avoid health care-related infections (9 questions), with a total of 41 items. Correct responses were assigned one score while incorrect /do not know responses assigned nil.
-For each domain of knowledge, the main source for information was inquired whether through the curriculum, self learning, ward practices or formal bed side teaching.
-Inquires exploring the attitudes of medical students towards the role current curriculum and teaching in providing essential training /orientation towards infection control and SPs, whether they are fulfilling their knowledge gap and their need for training in the domain of infection control and SPs were added. Five questions were adopted form the available literature (Humphreys & Richards, 2011) with Likert scale from strongly disagree to strongly agree.

### 2.4 Questionnaire Administration

Before questionnaire administration, investigators emphasized the right of the student of not to participate and the confidentiality of the process. Investigators conducted an orientation sessions for participants to explain the objectives, terms used in the data collection form and assist them for any difficulty if any.

Pilot testing was carried out on 3_rd_ year students (n=41), reliability analysis (Cronback's alpha) of the final data collection form (41 items) was .58, reliability coefficients for different components were as follow .51 for the general concept, hand hygiene= .74, PPE= .80, sharp injuries and disposal= .78, care of the HCPs= .81. The attitudes items showed a reliability coefficient of .72.

### 2.5 Data Analysis and Management

Data entered and analyzed using SPSS version 16.0 (Chicago, Ill, USA). Forms with missing of two or more items were discarded (n=14). Out of the total students invited (n=435), 265 responded (response rate of 60.9%). Fourth year (both genders n=142) showed a response rate of 57.0%, 5_th_ year (n=133) 62.4%, and 6_th_ year (n=145) 60.0%. Both descriptive and inferential statistical analyses were applied. For categorical variables, proportion and percentage were used for expression, Chi square and *Z* tests were used for comparison, for numerical variables, median, mean and standard deviation were employed for expression, Mann Whitney and Kruskal Wallis statistical tests were used for comparison. Scores for different domains were reported with a 25_th_ percentile of 16 points (out of 41), 50_th_ percentile of 20 and the 75_th_ percentile (24 points); we employed 24 score as a cut off for sufficient knowledge for infection control and SPs. Logistic regression model was generated to determine the possible predictors of acceptable knowledge (dependent variable) against the possible independent variables including gender, year at college, previous receiving of training and teaching/educational materials regarding infection control and SPs and the attitudes scores. P value of < 0.05 was set as the level of statistical significance.

## 3. Results

### 3.1 Basic Characteristics and General Concept of Infection Control and Standard Precautions and Hand Hygiene

Of the included participants 48.6% were females, 6.0% mentioned the receiving of previous training/orientation on infection control and SPs, and 21.5% had received teaching/educational materials regarding infection control and SPs (Table 1). The contents of the received training/orientation were in the form of half day sessions held at the local secondary level of care hospitals as a part of continuing medical education and composed of lectures and video demonstration of hand hygiene, sharp management and prevention of nosocomial infections.

**Table 1:** Characteristics of the medical students participated in the survey and correct responses about general concepts of infection control-standard precautions and hand hygiene by year of study

Characteristics	Years and Correct answers: No. (%)			

	Fourth (N=81)	Fifth (N=83)	Sixth (N=87)	Total (N=251)
**Gender**				
Males	40 (49.4)	43(51.8)	46(52.9)	129(51.4)
Females	41 (50.6)	40(48.2)	41(47.1)	122(48.6)
Previous training / orientation on SPs-IC	6(7.4)	4(4.8)	5(5.7)	15(6.0)
Received educational materials/instructions about SPs-IC	21(25.9)	17(20.5)	16(18.4)	54(21.5)
**General concept** **of infection control and standard precautions:**				
1- The main goal of infection control : **Options**	65(80.2)	59(71.1)	81(93.1)	205(81.7)
2- Definition of standard precautions: **Options**	33(40.7)	42(50.6)	48(55.1)	123(49.0)
3- All patients are sources of infection regardless their diagnoses. **True**	33(40.7)	31(37.3)	41(47.1)	105(41.8)
4- All body fluids except sweat should be viewed as sources of infection. **True**	25(30.9)	18(21.7)	37(42.5)	80(31.9)
5- All health providers are at risk of occupational infections. **True**	37(45.7)	54(65.1)	78(89.7)	189(75.3)
**Score for general concept (out of 5): Median (mean± SD)**	**3.0(2.6±0.9)**	**3.0(2.5±1.3)**	**4.0(3.6±1.0)**	**3.0(3.1±1.2)**
**Hand Hygiene:**				
1- Hand washing minimizes microorganisms acquired on the hands if soiled. **True**	42(51.9)	59(71.1)	62(71.3)	163(64.9)
2- Hand washing reduces the incidence of healthcare-related infections. **True**	53(65.4)	61(73.5)	65(74.7)	179(71.3)
3- Standard hand washing includes washing of both hands and wrists. **True**	53(65.4)	55(66.3)	59(67.8)	167(66.5)
4- In standard hand washing: minimum duration should be---. **Options**	34(42.0)	26(31.3)	38(43.7)	98(39.0)
5- Hand decontamination: includes washing the--with antiseptic soap for 30 seconds. **Options**	34(42.0)	35(42.2)	38(43.7)	107(42.6)
6- Alcohol hand rub substitutes hand washing even if the hands are soiled. **False**	22(27.2)	29(34.9)	21(24.1)	72(28.7)
7- Hand washing is indicated between tasks and procedures on the same patient. **True**	33(40.7)	37(44.6)	49(56.3)	119(47.4)
8- Use of gloves replaces the need for hand washing. **False**	31(38.3)	39(47.0)	41(47.1)	111(44.2)
9- Hand washing is indicated after removal of gloves. **True**	46(56.8)	46(55.4)	55(63.2)	147(58.6)
10- Hand washing is needed with patients with respiratory infections. **True**	26(32.1)	25(30.1)	35(40.2)	86(34.3)
**Score for hand hygiene (out of 10): Median (mean± SD).**	**5.5(5.0±2.1)**	**6.0(5.3±2.5)**	**6.0(6.2±2.1)**	**6.0(5.8±2.2)**

SPs: standard precautions, IC= infection control.

Table 1 also demonstrates the responses of knowledge items about the general concepts of infection control and SPs, 18.3% and 51% did not recognize the goal of infection control and the precise definition of SPs respectively. Only 41.8% recognized that all patients are sources of infection and only 31.9% stated that all body fluids except sweat should be viewed as sources of infection. For this domain a total score was 3.1±1.2 (out of 5), significantly higher among those at 6_th_ year (Kruskal Wallis, P=0.010). Only 39.0% were able to respond correctly about the standard duration of hand washing, 51.1% believed that alcohol hand rub can replace hand washing even if the hands were soiled, 33.5% acknowledged that using gloves replaces the need for hand washing and only 34.3% correctly stated the need for hand washing in dealing with patients with respiratory infections. For this domain a total knowledge score was 5.8±2.2 (out of 10 points), significantly more among those at 6_th_ year (P=0.013).

### 3.2 Personal Protective Equipment

Table 2 shows the correct responses to items related to the domain of Personal Protective Equipment (PPE) by clinical years included. Of the surveyed students 68.1% identified the role of PPE in complete elimination of the risk of acquiring infections, 74.5% stated that PPE should be exclusively used by laboratory and cleaning staff, and should be used only in presence of contact with blood (in 61.4%), 47.8% believed that gloves and masks should be re-used after proper cleaning. Also, 45.8% agreed that gloves should be changed between different procedures on the same patient, 43.4% correctly respond about re-using masks and gloves if dealing with the same patient. The total score for this domain was low (3.8±1.9 out of 9 points), and there was no statistical difference in relation to year of enrollment at the college.

**Table 2:** Correct responses about personal protective equipment by the included medical students by their years at college

Personal protective equipment	Years and Correct answers: No. (%)			

	Fourth (N=81)	Fifth (N=83)	Sixth (N=87)	Total (N=251)
1- PPE such as masks and head caps provides protective barriers against infection. **True**	42(51.9)	45(54.2)	49(56.3)	136(54.2)
2- Use of PPE eliminates risk of acquiring occupational infections. **True**	25(30.9)	25(30.1)	30(34.5)	80(31.9)
3- PPE is exclusively suitable to laboratory and cleaning staff for their protection. **False**	17(21.0)	20(24.1)	27(31.0)	64(25.5)
4- PPE should be used only whenever there is contact with blood. **False**	24(29.6)	31(37.3)	42(48.3)	97(38.6)
5- Gloves and masks can be re-used after proper cleaning. **False**	34(42.0)	46(55.4)	51(58.6)	131(52.2)
6- Used PPE are to be discarded through regular municipal disposal systems. **False**	24(29.6)	25(30.1)	21(24.1)	70(27.9)
7- Gloves should be changed between different procedures on the same patient. **True**	32(39.5)	38(45.8)	45(51.7)	115(45.8)
8- Masks made of cotton or gauze are most protective. **False**	14(17.3)	27(32.5)	25(28.7)	66(26.3)
9- Masks and gloves can be re-used if dealing with same patient. **False**	25(30.9)	30(36.1)	54(62.1)	109(43.4)
**Score for personal protective equipment: Median (mean± SD)**	**3.5(3.2±1.5)**	**4.0(3.7±2.2)**	**4.0(4.2±2.0)**	**4.0(3.8±1.9)**

PPE: Personal Protective Equipment.

### 3.3 Sharp Disposal, Sharp Injuries and Care of Health Providers

**Figure 1:**
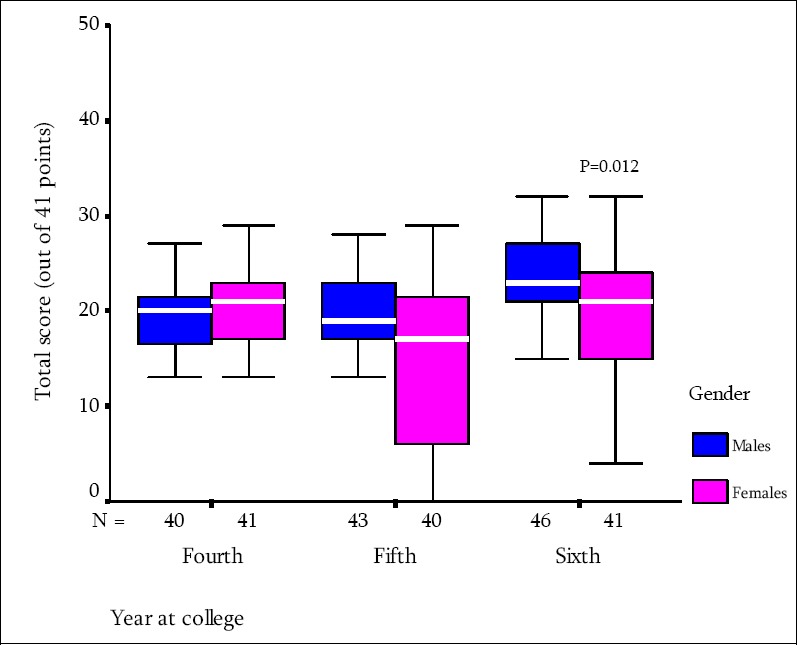
Total knowledge scores of the included medical students about standard precautions in relation tgenders and year at college, King Faisal University

Table 3 depicts the correct responses towards sharp disposal and sharp injuries and care of the health care providers. Only 17.9% and 32.7% respectively correctly responded to the false statements that used needles should be recapped or bent after use, more than 90% of the participants failed to identify the label of the container for sharp disposal. Also, 23.1% of the students correctly answered that post exposure prophylaxis is used in managing accidental sharp injuries from an HIV-infected patient and 22.7% correctly stated that the immediate management of sharp injuries including washing in running water and soap. The total score of this domain was 2.8±1.9 (out of 8 points). Those of 6_th_ years have significantly higher scores compared to 4_th_ and 5_th_ years (P=0.008). Care of health care providers showed that students’ responses reflecting their lack of knowledge, more than 75% failed to identify the required vaccinations for health care providers, only 21.1% of students correctly identified the size of risk following the exposure to needle stick injuries from an HIV/AIDS patient. The total score of this domain was 3.8±2.0 (out of 9 points), significantly more among 6_th_ year and higher among males (Figure 1).

**Table 3:** Correct responses of medical students about sharp injuries/disposal and health of health care providers by years of study

Sharps disposal and sharp injuries	Years and Correct answers: No. (%)			

	Fourth (N=81)	Fifth (N=83)	Sixth (N=87)	Total (N=251)
1- Used needles should be recapped after use to prevent injuries. **False**	9(11.1)	16(19.3)	20(23.0)	45(17.9)
2- Used needles should be bent after use to prevent injuries. **False**	18(22.2)	28(33.7)	36(41.4)	82(32.7)
3- Sharps container is labeled with …. : **Options**	3(3.7)	8(9.6)	7(8.0)	18(7.2)
4- Soiled sharps objects should be shredded before final disposal. **True**	16(19.8)	19(22.9)	21(24.1)	56(22.3)
5- Sharps injuries should be managed with no need of reporting. **False**	22(27.2)	27(32.5)	31(35.6)	80(31.9)
6- Needle-stick injuries are the least commonly encountered in general practice. **False**	30(37.0)	31(37.3)	42(48.3)	103(41.0)
7- Post-exposure prophylaxis is used for managing injuries from an HIV-infected patient. **True**	16(19.8)	17(20.5)	25(28.7)	58(23.1)
8- Immediate management of sharps injuries includes: **Options**	14(17.3)	23(27.7)	20(23.0)	57(22.7)
**Score for sharps disposal and injuries: Median (mean± SD).**	**3.0(2.7±1.5)**	**3.0(2.6±2.0)**	**3.0(3.4±1.9)**	**3.0(2.8±1.9)**
**Care of healthcare providers:**				
1- Immunization history of health care providers should be obtained before recruitment. **True**	37(45.7)	46(55.4)	56(64.4)	139(55.4)
2- Routine immunizations for healthcare providers include HIV, rubella and rabies. **False**	15(18.5)	20(24.1)	27(31.0)	62(24.7)
3- Healthcare providers should receive annual influenza vaccine. **True**	29(35.8)	32(38.6)	45(51.7)	106(42.2)
4- Healthcare providers should be tested annually by tuberculin skin test. **True**	24(29.6)	23(27.7)	32(36.8)	79(31.5)
5- The risk for a health provider to acquire HIV infection after needle-stick injury is: **Options**	16(19.8)	17(20.5)	20(23.0)	53(21.1)
6- Post exposure immunization prevents the risk of hepatitis B infection following exposure. **True**	32(39.5)	28(33.7)	48(55.2)	108(43.0)
7- For the prevention of hepatitis B, immunizations are recommended for all healthcare workers. **True**	52(64.2)	51(61.4)	55(63.2)	158(62.9)
8- Following exposure to a patient with flu, antibiotics are required for prevention of infection. **False**	12(14.8)	18(21.7)	22(25.3)	52(20.7)
9- Health providers with highest risk of exposure to tuberculosis include radiologists. **True**	16(19.8)	25(30.1)	31(35.6)	72(28.7)
**Score for care of the healthcare workers: Median (mean± SD).**	**4.0(4.0±1.5)**	**3.0(3.1±2.2)**	**5.0(4.3±2.3)**	**4.0(3.8±2.0)**

### 3.4 The Acceptable Level of Knowledge and Its Correlates

We have set the cutoff for being knowledgeable towards infection control and SPs with scores that ≥ the 75^th^ percentile (≥ 24 out of a total of 41 points), of the total, 67 (26.7%) students scored ≥ 24, 18/81 (22.2) in 4^th^ year, 17/83 (20.5%) in 5^th^ year and 32/87 (36.8%) in 6^th^ year, 37/129 (28.7%) among males and 30/122 (24.6%) among females (P=0.463). Table 4 depicts the results of binary logistic regression analysis, knowledge score was significantly positively correlated with the year at the college and significantly negatively associated with non receiving of teaching/educational training materials, while gender, receiving of previous training/orientation towards infection control and SPs and attitude score had no significant association with the knowledge scores.

**Table 4:** Logistic regression model for the possible correlates of higher knowledge toward standard precautions among the included medical students

Independent variables	B	S.E.	P value	Odds ratio	95% Confidence intervals	
**Gender (female)**	-.471	.305	0.123	0.62	(0.34-1.14)	
**Year at the college**	.415	.195	0.034	1.51	(1.03-2.22)	
**Previous training on infection control/SP (no)**	-.499	.493	0.310	0.61	(0.23-1.60)	
**Received educational materials/instructions on IC/SP (no)**	-.682	.330	0.039	0.51	(0.27-0.97)	
**Attitude scores**	.070	.061	0.251	1.072	(0.95-1.21)	

B= régression coefficient, S.E= standard error.

Percent predicted =71.4, Hosmer and Lemeshow Chi square for the model=8.65, P=0.061.

### 3.5 Sources of Students’ Knowledge

**Figure 2:**
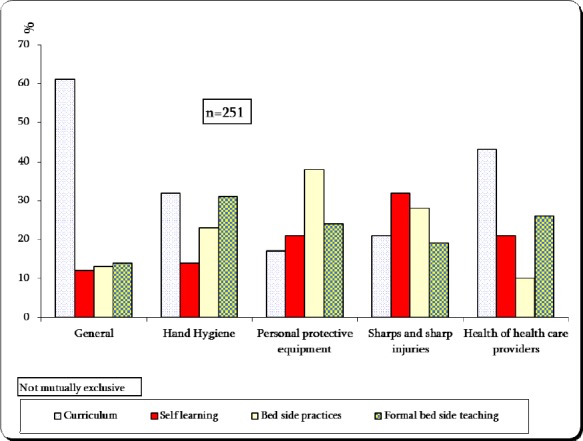
The main sources for information about the different domains of infection control and standard precautions among the surveyed medical students at King Faisal University

Figure 2 shows the main sources of medical students’ information in relation to the different domains of infection control and SPs. With the exception of the general concept and health care of health providers’ domains, the current curriculum was not cited as the main source of their knowledge especially for the PPE and sharps management domains.

### 3.6 Attitudes toward the Received Training/Teaching of Infection Control and SPs

Table 5 depicts the attitudes of medical students towards their satisfaction with the current curricular content and the received training towards infection control and SPs. Of the included students 61.4% disagreed and strongly disagreed that the current curriculum provides them with enough information on infection control and SPs, 69.9% disagreed and strongly disagreed about the availability of extracurricular training and/or orientation sessions towards infection control and SPs at the college, 60.1% disagreed and strongly disagreed about the role of their tutors and faculty in providing them with necessary information on how to avoid health facilities related infections before their entrance into clinical training at hospitals, and almost 80% of the included students agreed or strongly agreed about their need to receive training and orientations towards infection control and SPs. The attitude score showed a total of 9.6±2.7 (median of 9.0 out of 15 points), 4^th^ year attitude score was the least (8.5±2.6, compared to 9.3±2.2, 11.0±2.7 for the 5^th^ and 6^th^ years respectively (Kruskall Wallis, P=0.001).

**Table 5:** Attitudes of the included medical students towards current curricular sufficiency and their training needs for infection control and standard precautions

Responses: No. (%)					Statements

Strongly disagree	Disagree	Neutral	Agree	Strongly agree	
68(27.1)	86(34.3)	41(16.3)	40(15.9)	16(6.4)	1- Current curriculum provides enough information on IC and SPs.
99(39.4)	74(29.5)	39(15.5)	24(9.6)	15(6.0)	2- Training/orientation sessions about IC and SPs are provided to medical students.
101(40.2)	50(19.9)	58(23.1)	31(12.3)	11(4.4)	3-Tutors and faculty provided us with enough information on how to avoid health facilities related- infections before clinical rotations.
94(37.5)	77(30.7)	43(17.1)	19(7.6)	18(7.2)	4- I received hands on training on how to avoid health facilities-related infections using case scenarios and simulations.
5(2.0)	6(2.4)	40(15.9)	111(44.2)	89(35.5)	5- I need to receive training on IC and SPs.

IC= infection control, SPs= standard precautions

## 4. Discussion

In this study the total score for knowledge was 19.3±9.1 (out of 41 points) with a total of 67 students (26.7%) scored ≥ 24 which is considered to be acceptable, students’ knowledge differed according to the specific areas, the highest scores was noticed along the domain of hand hygiene, and care of the health care providers, while sharp management and injuries and PPE showed the least scores. Tavolacci et al. (2008) reported in their study that the highest scores were achieved for knowledge of standard precautions and hand hygiene, and the worst score was for knowledge of nosocomial infection. Knowledge of hand hygiene should be improved, because it is the most effective measure for interrupting the transmission of microorganisms that cause nosocomial infection (Tavolacci et al., 2008), interventions are needed to increase the knowledge in the domains of sharp management and injuries and PPE among our students.

Also, in this study, 18.3% and 51% did not recognize the goal of infection control and the precise definition of SPs respectively, and only 41.8% recognized that all patients irrespective of their diagnoses are sources of infection. These findings are consistent with the results of Amin & Al Wehedy (2009) who found a similar pattern among primary care providers in Al Hassa, Saudi Arabia.

For hand hygiene, only 39.0% were able to respond correctly about the standard duration of hand washing, 51.1% believed that alcohol hand rub can replace hand washing even if the hands were soiled, 33.5% incorrectly acknowledged that using gloves replaces the need for hand washing and only 34.3% correctly stated the need for hand washing in dealing with patients with respiratory infections. Students in this domain demonstrated an acceptable level of knowledge as 156 (62.2%) scored 6 or more points (out of 10). These results are compatible with findings reported from different colleges in the Middle East and Western countries (Askarian et al., 2004; Barikani, 2012; Tavolacci et al., 2008; Mann & Wood 2006; Koeing & Chu, 1993; Kim et al., 2001).

Askarian et al. (2004) have found that despite of the acceptable knowledge of medical students, there was poor compliance especially in hand hygiene, others added that while it seems rational that knowledge and attitude should have an impact on practice, no change will be observed if it is not possible to comply with existing recommendations, and the found disparity between knowledge and practice could also be due to the unavailability of protective barriers, inadequate equipment, carelessness, malpractice of senior colleagues or interference of devices with working skills (Kim et al., 2001).

The results of this study endorsed the need for curricular changes as recommended by similar study among medical students in Qassim Medical school, Saudi Arabia to assess their knowledge and compliance with hand hygiene, where it was found that only 29% of students were able to identify all the five indications related to hand hygiene, with overall hand hygiene compliance of only 17% (al-Kadi & Salatai, 2012). Lack of hand hygiene awareness and compliance in medical students has been attributed to many factors. The most important is the lack of role model; the behavior of students is strongly influenced and molded by their mentor's attitude at the bed side. This has been validated in reference to hand hygiene practices in multiple studies (Mann & Wood, 2006; Koeing & Chu, 1993). The role models change with each passing year of training from teachers to senior colleagues and if any of these role models are performing faulty hand hygiene, as is very common in hospital settings, and then the students are likely to be less compliant (al-Kadi & Salati, 2012).

The current study revealed that many misconceptions related to the PPE indications, uses, and their role in preventing nosocomial infections are present. This domain showed the least level of knowledge with several misconceptions. The included students showed a much lower score than those reported from Western similar studies where it was found that for instance 65% of medical students gave the correct answers about the use of gloves (Jeffe et al., 1998). Further, this study also showed that only 17.9% and 32.7% respectively provided correct responses about pending and recapping of the used needles, only 23.1% of the students correctly mentioned the role of post exposure prophylaxis in managing sharp injuries from an HIV-infected patient and 22.7% correctly stated that the immediate management of sharp injuries includes washing in running water and soap. Similar results were found among health care providers (HCPs) at the primary level of care in Saudi Arabia (Amin & Al Wehedy, 2009).

Kim et al. (2001) reported that knowledge of SPs especially in the domain of sharp injuries was better among nursing students than among medical students. Nurses are a subgroup of HCPs at high risk for needle stick injuries because they are frequently involved with invasive procedures. However, it could be expected that the knowledge level about infection control and SPs would be similar regardless of curriculum. Medical students have just as high a risk of needle stick injuries as do nursing students, because of their limited clinical experience, (Feather et al., 2000; Monsalve et al., 2007; Smith & Leggat, 2006) and they should observe the same precautions during patient care to avoid cross-transmission and to prevent spread of nosocomial infection.

In this study, only 26.7% of the surveyed students were found to have an acceptable level of knowledge. The previous notion implying that students who are lacking the proper knowledge and if this is coupled with presence of misconceptions especially related to hand hygiene, sharp management and PPE, this combination will make them more vulnerable to health care facilities - related infections. These results are consistent with those reported from other medical schools in the Middle East where the overall knowledge for SPs is poor (Askarian et al., 2004; al-Kadi & Salati, 2012).

In this study, self learning and informal bed side clinical practices were the main sources for knowledge as cited by many students especially in domains of sharp injuries, PPE and care of health care providers, these results are inconsistent with studies carried out in more developed countries where teaching during the curriculum was the main source of information, and the information about SPs was emphasized more during the curriculum for nursing students than for the medial students (Tavolacci et al., 2008).

In addition, the study also demonstrated the attitudes of medial students regarding the current teaching and curricular contents for infection control and SPs with substantial perception that the current curriculum, the received training and orientation at the clinical teaching are insufficient which endorse the fore mentioned findings about the main sources of information among the surveyed students.

Learning practices are indispensable for improving student knowledge of nosocomial infection (Rosethal et al., 1999; Snow et al., 2006) and the prevention of infection transmission (Calabro et al., 1998; Jeffe, 1999; Deisenhammer et al., 2006). In this study, the level of knowledge was significantly correlated with year at college; this can be explained by the fact that those at advanced years are more exposed to clinical practices with substantial exposures to patients, clinical practices and senior clinical staff in hospital wards compared to 4^th^ and 5^th^ years. Teaching infection control to health care students is a challenge both with respect to developing a cohesive program and encouraging students to adopt correct attitudes early in their careers. Kim et al. (2001) showed a positive correlation between knowledge and performance of SPs. Nobile et al. (2002) reported that a positive attitude about hand disinfection was higher among HCPs with a higher level of knowledge. However, educational background is one of the factors influencing compliance with good practices and education works synergistically with other factors namely behavior and practice (Nobile et al., 2002; Pittet, 2004; Helfgott et al., 1998; Jumaa, 2005). In this study previous training on SPs and infection control was not a positive predictor for higher knowledge and this could be referred to nature and contents of these training (limited in time and contents, mostly non specific and without hands on training). Studies showed that specific training of SPs can quickly improve students’ knowledge of infection control in a short period of time (Colombo et al., 2002; Nobile et al., 2002; Diekema et al., 1995). Some authors recommended that future educational approaches should include rigorous curricular reform with pragmatic presentation of effective hand hygiene and SPs, feedback from teachers at the bedside, and inclusion of hygiene scores for students in all clinical training courses (Tavolacci et al., 2008; Kim et al., 2001).

## 5. Study Limitations

The study inherent a study design limitation for being a cross-sectional, secondly, observation of students’ behaviors, skills and compliance was not possible as the clinical rotations were carried out at facilities not affiliated to the College of Medicine and finally, the possible problem with the questionnaire design as most of the items had the options of true/false which may provide the opportunity for guessing.

## 6. Conclusion

The overall knowledge scores for SPs were low especially in the domains of hand hygiene, sharp management, and personal protective equipment reflecting insufficient and ineffective instructions received by medical students through the current curriculum posing them vulnerable to health facilities related infections. Proper curricular reform and training are required to protect students and their patients.
